# Effects of *Ex-Vivo* and *In-Vivo* Treatment with Probiotics on the Inflammasome in Dogs with Chronic Enteropathy

**DOI:** 10.1371/journal.pone.0120779

**Published:** 2015-03-23

**Authors:** Silke Schmitz, Dirk Werling, Karin Allenspach

**Affiliations:** 1 Department of Veterinary Sciences and Services, Royal Veterinary College, University of London, Hawkshead Campus, North Mymms, United Kingdom; 2 Department of Pathology and Pathogen Biology, Royal Veterinary College, University of London, Hawkshead Campus, North Mymms, United Kingdom; University of Iowa Carver College of Medicine, UNITED STATES

## Abstract

Inflammasomes coordinate the maturation of IL-1β and IL-18 in response to danger signals. They are vital for maintenance of intestinal homeostasis and have been linked to chronic intestinal inflammation in humans. Probiotics have been advocated as treatment in intestinal inflammation. So far, no study has investigated the role of the inflammasome in canine chronic enteropathy (CE). In this study the intestinal expression of inflammasome components was assessed in CE dogs compared to controls, when treated with probiotic *Enterococcus faecium* (EF) *ex-vivo* and *in-vivo*. RNA extraction from endoscopic biopsies and reverse-transcriptase quantitative PCR was performed for NLRP3, casp-1, IL-1β and IL-18. Immunohistochemistry was performed to investigate protein expression in tissues. Gene expression of casp-1 and NLRP3 was lower in CE samples than controls. *Ex-vivo* treatment with EF reduced NLRP3 expression in control samples. Treatment of CE dogs with EF alongside dietary intervention had no effect on gene expression. In contrast, IL-1β protein expression in CE decreased with dietary treatment (but not with probiotics). The results of this study suggest that the inflammasome or its components may be partially involved in the inflammatory process seen in CE, but distinct from intestinal inflammation in humans.

## Introduction

In dogs, chronic enteropathies (CE) are a group of disorders of unknown cause associated with chronic gastrointestinal (GI) symptoms of unknown cause [1]. There are parallels to human Inflammatory Bowel Disease (IBD), as both are considered chronic immune-mediated inflammatory diseases of the GI tract that result from a dysregulated mucosal immune response to bacterial antigens [2]. Inflammasomes are found in a wide range of cell types including macrophages, dendritic cells and intestinal epithelial cells [3]. The inflammasome complex is composed of multiple cytosolic proteins, including NOD-like receptors (NLRs), the adaptor protein ASC (apoptosis-associated speck-like protein containing a CARD domain) and caspase-1 (casp-1). NLRs bind to bacterial constituents, purine-like compounds, and other substrates [4–7]. They are defined by their N-terminal region, which most commonly holds a caspase-recruitment domain (CARD) or a pyrin domain (PYD) [8]. Depending of the presence of these domains, the receptors are classified into either NLRCs (NLR with CARD) or NLRPs (NLR with PYD). Activation of both types of receptors results in casp-1 activation, which cleaves the precursors of the inflammatory cytokines Interleukin (IL)-1β and IL-18 into their active proteins [7], whereas IL-33 becomes inactivated [9]. Secretion of the bioactive cytokines enhances antimicrobial functions of innate immune cells, promotes protection against intracellular pathogens [10, 11], and can elicit pyroptosis (inflammatory cell death) of the inflammasome-activated cell [12].

Results of several studies point towards a potential role of inflammasomes in the development of chronic intestinal inflammation. In human patients with IBD, both IL-1β and IL-18 are up-regulated on the mRNA and protein level in the colon [13–16]. Furthermore, genetic variations of IL-18 and NLRP3 have been associated with the development of human Crohn’s disease [17, 18], whereas an involvement of the NLRP3 inflammasome in the development of ulcerative colitis (UC) is still a matter of debate. NLRs, especially NLRP3 play a dual and somewhat contradictory role in the intestine: NLRP3 seems to be partly responsible for maintaining mucosal barrier homeostasis as it protects from epithelial injury, as some studies have shown that mice lacking NLRP3 are more susceptible to develop chemically induced colitis, similar to mice lacking ASC or casp-1. However, other studies showed that colitis in rodents is actually mediated by NLRP3 [3, 19, 20]. As most of the functions of NLRP3 seem to be driven by microbial signals from the commensal microbiota, differential functions of the inflammasomes depending on distinct microbial signalling is possible [21, 22].

Because NLRs recognise bacterial components, they have an important function in the cross-talk between the intestinal microbiota and the local immune system. Hence, the application of “beneficial” microbes might provide an opportunity to improve inflammatory intestinal conditions, such as CE or IBD. These probiotics have been defined by the World Health Organisation to be “live microbes which, when administered in adequate amounts, confer a health benefit to the host” [23]. They are frequently commensal bacteria, but can also be sourced from fermented foods or the environment [24]. Several studies have shown that probiotics can influence key biological signalling pathways of inflammation, which can be specific to an individual probiotic strain, including strains of the same bacterial species [25].

There is not much known about the function and expression of inflammasomes in the canine gut, and their role in canine CE is unexplored. So far, one study investigated IL-1β and the IL-1β receptor antagonist in canine IBD tissues compared to tissues from healthy control animals and found an altered ratio of those proteins in samples from IBD cases. However, there was no absolute change in IL-1β mRNA or protein in this study, which possibly indicates an increase in inflammatory signals driven by IL-1β in canine IBD [26].

The purpose of the present study was to examine the role of different components and outputs of the inflammasome (NLRs, casp-1, IL-1β, IL-18) in canine CE and determine if the expression of these components is influenced by the *ex-vivo* and *in-vivo* treatment with a probiotic bacterial strain.

## Results

### NLRP3, but not NLRP1 or NLRC4 are expressed in healthy canine intestinal tissues

**Fig 1:**
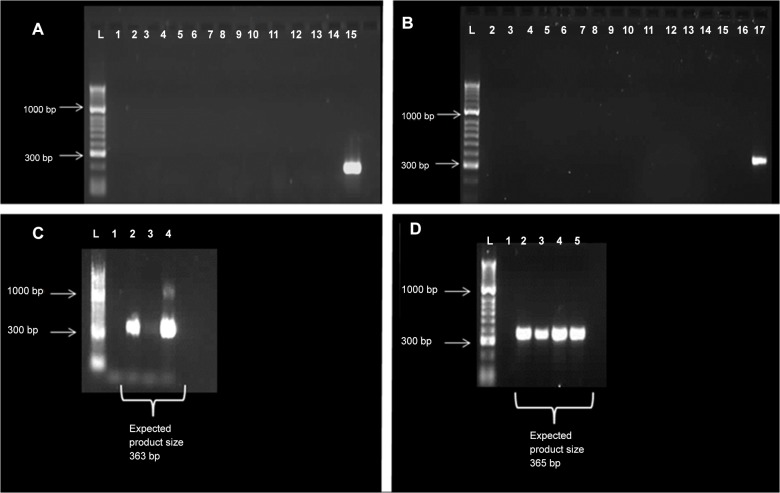
Amplification of NLRP1 and NLRC4 genes from canine samples. Both NLRP1 (A,C; (expected product size 363 bp) and NLRC4 (B,D; expected product size 365 bp) expression could not be detected in cDNA (A, B), but could be in gDNA (C, D). L = hyperladder II (Bioline, UK). All labelled lanes (exception lane 1 = empty) have been loaded with cDNA or gDNA samples, respectively. The last lane in A (lane 15) and B (lane 17) are an assay control with GAPDH amplification from a cDNA sample (product size 194 bp).

**Fig 2:**
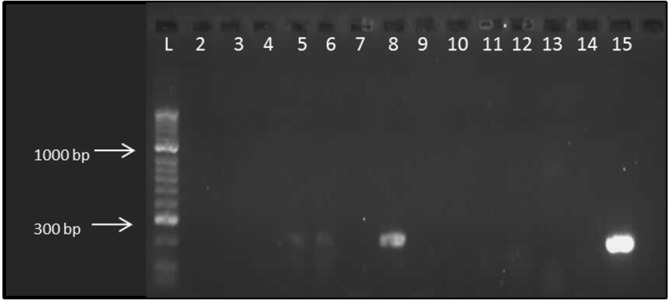
Amplification of NLRP3 from canine intestinal cDNA samples (expected product size 207 bp). All labelled lanes have been loaded with cDNA. Lane 15 is an assay control with GAPDH on an intestinal cDNA sample (product size 194 bp). L = hyperladder II (Bioline, UK).

Initially, the expression of several NLRs involved in formation of inflammasomes was examined in canine intestinal tissue by standard PCR. For this, cDNA derived from the duodenum and colon of 7 healthy Beagle dogs and 6 dogs with CE was used (their signalment can be found in Table 1). No mRNA transcripts for NLRP1 or NLRC4 were detectable in any sample (Fig. 1); despite the fact that gDNA for these genes was readily detectable (Fig. 2). In contrast, mRNA transcripts for NLRP3 were detected in intestinal cDNA samples from some healthy dogs (Fig. 1). Based on these initial findings, it was decided to quantify mRNA expression of NLRP3 together with IL-1β, IL-18 and casp-1 in a selection of intestinal samples taken from healthy and diseased dogs.

**Table 1:** Signalment of healthy control dogs (C) and dogs with chronic enteropathy (CE) from which duodenal (duo) and colonic (col) samples were used for assessing gene and protein expression of inflammasome-components.

**sample ID**	**breed**	**age (months)**	**gender**	**sample type**	**used for**
CE1	Labrador	29	f	duo	baseline gene expression
CE2	Bichon Frise	87	mn	duo	baseline gene expression
CE3	Boxer	27	mn	duo	baseline gene expression
CE4	Weimaraner	71	fn	duo	baseline gene expression
CE5	Golden Retriever	14	f	duo	baseline gene expression
CE6	Cross breed	50	mn	duo	baseline gene expression
CE7	CKCS	64	f	duo	comparison healthy to CE, IHC
CE8	Golden Retriever	139	m	duo	comparison healthy to CE, IHC
CE9	Rottweiler	66	m	duo	comparison healthy to CE, IHC
CE10	Tibetan Spaniel	85	m	duo	comparison healthy to CE, IHC
CE11	English Pointer	156	fn	duo	comparison healthy to CE, IHC
CE12	Yorkshire Terrier	24	mn	duo	comparison healthy to CE, IHC
CE13	Cocker Spaniel	129	fn	duo	comparison healthy to CE, IHC
CE14	Golden Retriever	108	mn	duo	comparison healthy to CE, IHC
CE15	Rottweiler	30	m	duo	comparison healthy to CE, IHC
CE16	Gross breed	11	m	duo	comparison healthy to CE, IHC
CE17	Boxer	159	mn	duo	comparison healthy to CE, IHC
CE18	Labrador	48	f	duo	comparison healthy to CE
CE19	CKCS	12	fn	duo	comparison healthy to CE
CE20	Weimaraner	131	fn	duo	comparison healthy to CE
CE21	CKCS	131	fn	duo	comparison healthy to CE
CE22	Cross breed	73	mn	duo	comparison healthy to CE
CE23	Basset Hound	41	m	duo	comparison healthy to CE
CE24	Yorkshire Terrier	20	m	duo	comparison healthy to CE
CE25	Cross breed	76	m	duo	comparison healthy to CE
CE26	Rhodesian Ridgeback	139	fn	duo	comparison healthy to CE
CE27	Cairn Terrier	89	fn	duo	comparison healthy to CE
CE28	Staffordshire	76	m	duo	comparison healthy to CE
CE29	Labrador	33	m	duo, col	clinical trial, ex-vivo, IHC
CE30	Standard Poodle	67	m	duo, col	clinical trial, ex-vivo, IHC
CE31	Labrador	46	fn	duo, col	clinical trial, ex-vivo, IHC
CE32	Labrador	13	mn	duo, col	clinical trial, ex-vivo, IHC
CE33	Miniature Schnauzer	78	m	duo, col	clinical trial, ex-vivo, IHC
CE34	Golden Retriever	24	m	duo, col	clinical trial, ex-vivo, IHC
CE35	English Setter	18	mn	duo, col	clinical trial, ex-vivo, IHC
CE36	Labrador	49	fn	duo, col	clinical trial, ex-vivo, IHC
CE37	Golden Retriever	66	fn	duo, col	clinical trial, ex-vivo, IHC
CE38	Labrador	29	m	duo, col	clinical trial, ex-vivo
CE39	Bracco Italiano	12	m	duo, col	clinical trial, ex-vivo
CE40	Labrador	84	fn	duo, col	clinical trial, ex-vivo
CE 41	Cockapoo	39	fn	duo	ex-vivo
CE42	Labrador	98	fn	duo	ex-vivo
CE43	Golden Retriever	55	fn	duo	ex-vivo
CE44	Boxer	40	mn	duo	ex-vivo
CE45	Golden Retriever	8	mn	duo	ex-vivo
C1	Beagle	131	m	duo	baseline gene expression
C2	Beagle	49	m	duo	baseline gene expression
C3	Beagle	31	f	duo	baseline gene expression
C4	Beagle	43	f	duo	baseline gene expression
C5	Beagle	56	f	duo	baseline gene expression
C6	Beagle	17	m	duo	baseline gene expression
C7	Beagle	43	f	duo	baseline gene expression
C8	Greyhound	61	fn	duo	comparison healthy to CE
C9	Greyhound	48	fn	duo	comparison healthy to CE
C10	Greyhound	72	m	duo	comparison healthy to CE
C11	Greyhound	48	f	duo	comparison healthy to CE
C12	Greyhound	36	fn	duo	comparison healthy to CE
C13	Beagle	36	m	duo	comparison healthy to CE
C14	Beagle	24	m	duo	comparison healthy to CE
C15	Beagle	48	fn	duo	comparison healthy to CE
C16	Beagle	60	m	duo	comparison healthy to CE
C17	Beagle	36	f	duo	comparison healthy to CE
C18	Beagle	36	m	duo	comparison healthy to CE
C19	Beagle	96	f	duo	comparison healthy to CE
C20	Beagle	12	f	duo	comparison healthy to CE
C21	Beagle	24	m	duo	comparison healthy to CE
C22	Beagle	36	m	duo	comparison healthy to CE
C23	Beagle	16	f	duo	comparison healthy to CE
C24	Beagle	32	m	duo	comparison healthy to CE
C25	Beagle	48	f	duo	comparison healthy to CE
C26	Beagle	21	f	duo	ex-vivo, IHC
C27	Beagle	24	f	duo	ex-vivo, IHC
C28	Beagle	23	f	duo	ex-vivo, IHC
C29	Beagle	33	f	duo	ex-vivo, IHC
C30	Beagle	30	m	duo	ex-vivo, IHC
C31	Beagle	33	m	duo	ex-vivo, IHC
C32	Beagle	24	f	duo	ex-vivo, IHC
C33	Beagle	41	f	duo	ex-vivo, IHC
C34	Beagle	35	f	duo	ex-vivo, IHC
C35	Beagle	23	f	duo	ex-vivo, IHC
C36	Beagle	32	f	duo	ex-vivo, IHC
C37	Beagle	95	f	duo	IHC

Most of these samples were part of a tissue archive, whereas samples from the clinical trial were collected prospectively. F = female, fn = female neutered, m = male, mn = male neutered, IHC = immunohistochemistry, ex-vivo = ex-vivo tissue stimulation with different ligands.

### Expression of the NLRP3 and caspase-1 mRNA is reduced in samples from dogs with chronic enteropathy

**Fig 3:**
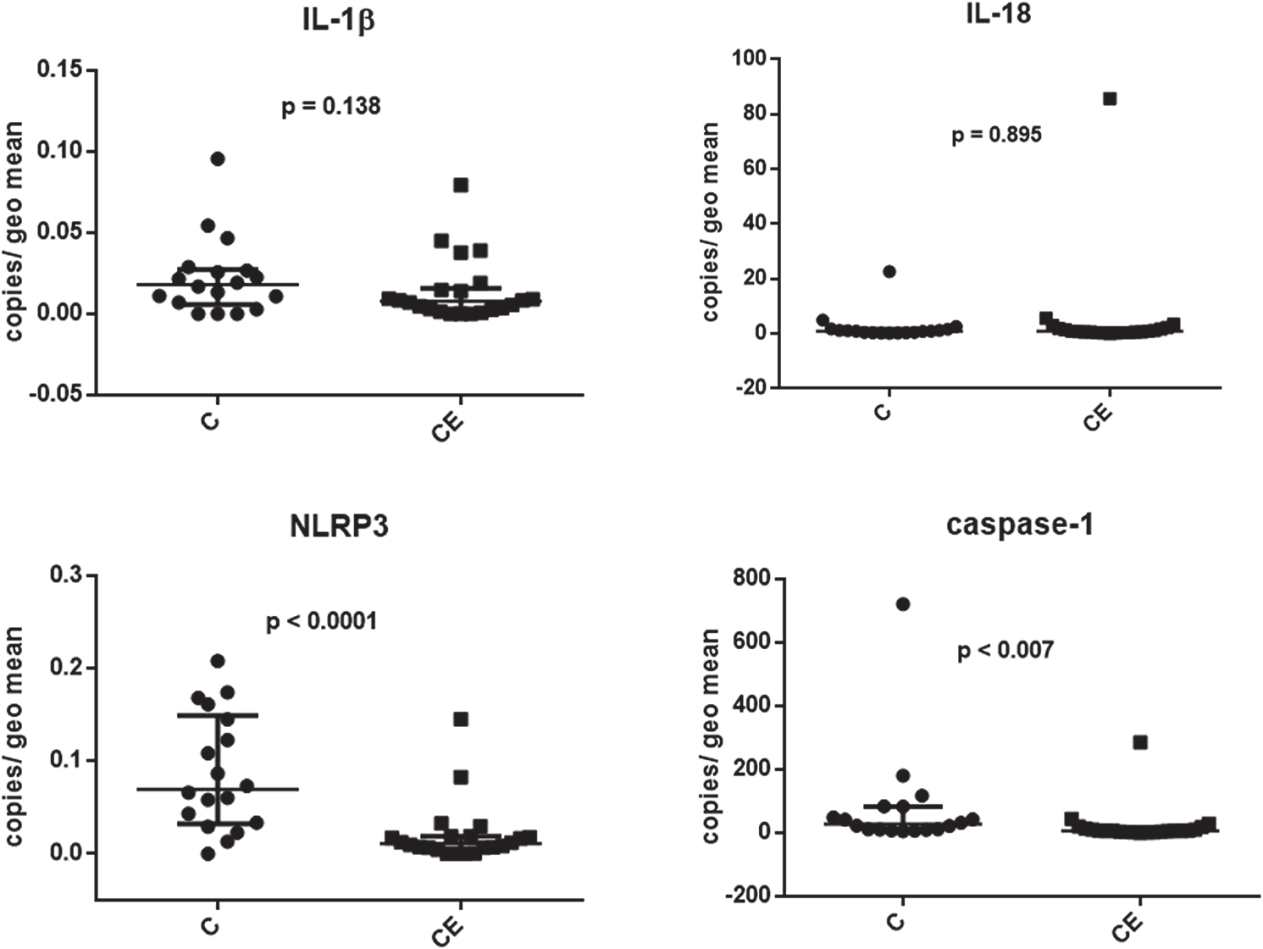
Relative expression of inflammasome-related genes in canine duodenal tissue. Expression levels from healthy control dogs (C) and dogs with chronic enteropathy (CE) were compared using Mann Whitney U test. Data are presented as median and interquartile range.

Having established which NLRs and down-stream molecules can be detected by RT-PCR, we next assessed the expression of mRNA transcripts for NLRP3, IL-1β, IL-18 and casp-1 by qPCR in duodenal samples taken from 18 healthy and 22 dogs with CE (their signalment and clinical data can be found in Table 1). Expression levels for casp-1 and NLRP3 mRNA were significantly lower in samples form dogs with CE compared to healthy controls (Fig. 3). In contrast, there was no significant difference in mRNA expression levels for IL-1β and IL-18 between the groups.

### *Ex-vivo* stimulation with probiotic Enterococcus faecium increases caspase-1 mRNA expression compared to other TLR ligands, but has no influence on other inflammasome genes

**Fig 4:**
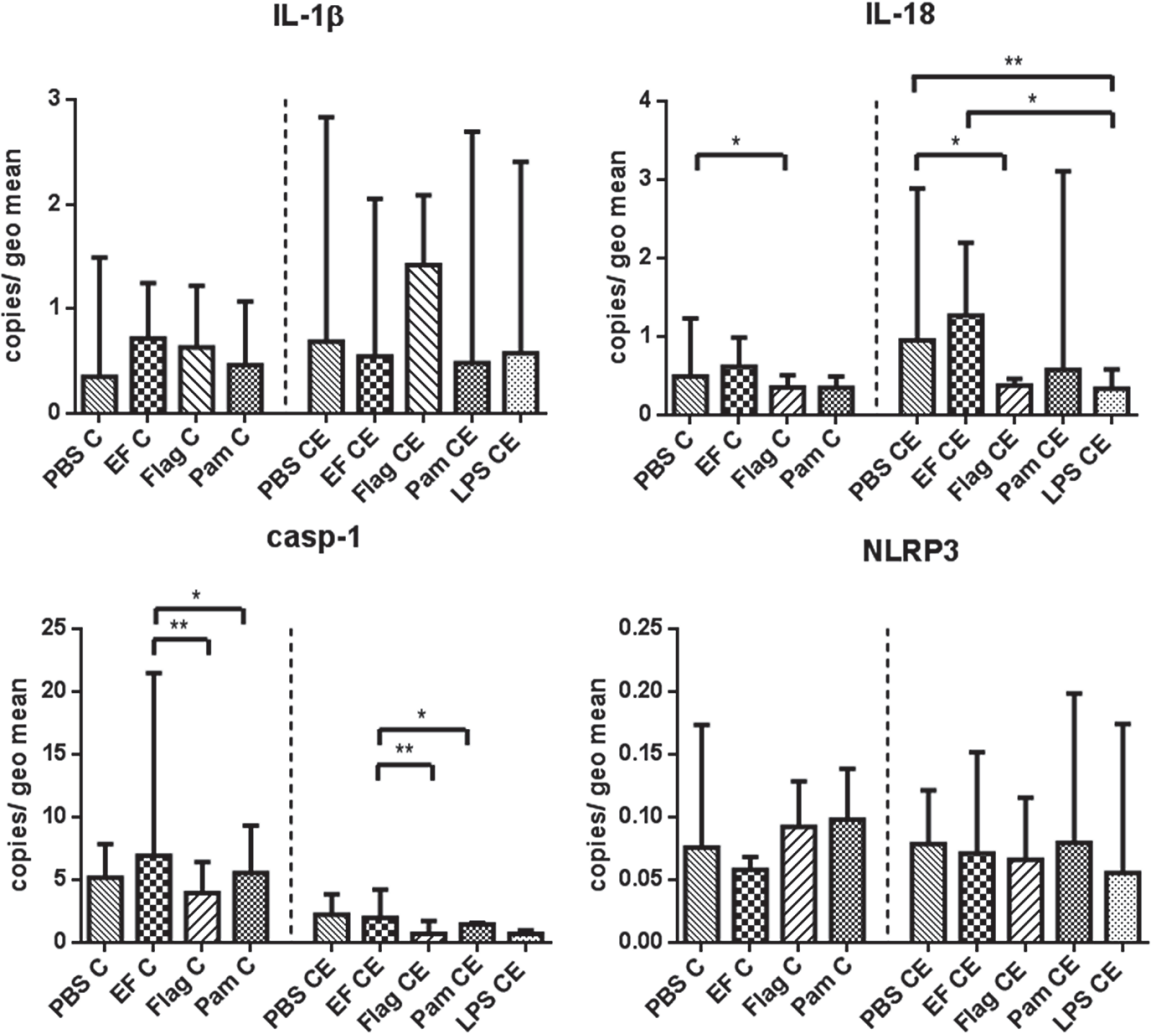
Expression of inflammasome-related genes in duodenal samples stimulated with different Toll-like receptor (TLR) ligands and *Enterococcus faecium* NCIMB 10415 (EF). Endoscopic biopsies were incubated for 5 hours with the respective stimulant. PBS = phosphate buffered saline (control), Flag = flagellin (TLR5 ligand), Pam = Pam_3_CSK_4_ (TLR1/2 ligand), LPS = lipopolysaccharides (TLR4 ligand). Linear mixed modelling was performed to compare data; using both “treatment” as well as “disease status” as a main effect. IL-18 decrease in response to LPS or flagellin is different between samples from diseased and healthy dogs, whereas the casp-1 increase seen with EF stimulation is independent of disease status. Data are presented as median with 10–90 percentiles.* p < 0.05, ** p < 0.01.

Having established differences in NLRs expression in biopsies from healthy dogs and dogs with CE, we next wanted to know whether e*x-vivo* stimulation of intestinal biopsies with EF impacts on NLR mRNA expression levels. To do so, fresh endoscopic duodenal biopsies were taken from 17 dogs with CE and 11 healthy controls (their signalment can be found in Table 1). Stimulation included culturing the biopsies in cell culture medium and respective additives (TLR-ligands, live EF or PBS as a control) for 5 hours at standard culture conditions (see below). The stimulation induced significant changes in expression of some inflammasome-related genes (Fig. 4). IL-18 mRNA expression was significantly down-regulated when intestinal samples from CE dogs were stimulated with LPS or flagellin compared to mRNA expression levels in samples from controls. Casp-1 mRNA expression was significantly increased upon stimulation with EF independent of the disease-status when compared to stimulation with single TLR ligands, but was not different compared to PBS. mRNA expression of IL-1β and NLRP3 was not significantly different with regards to disease status or across different treatments in the full mixed-model analysis.

### IL-1β protein production is increased in inflamed duodenal tissue and reduces with treatment

**Fig 5:**
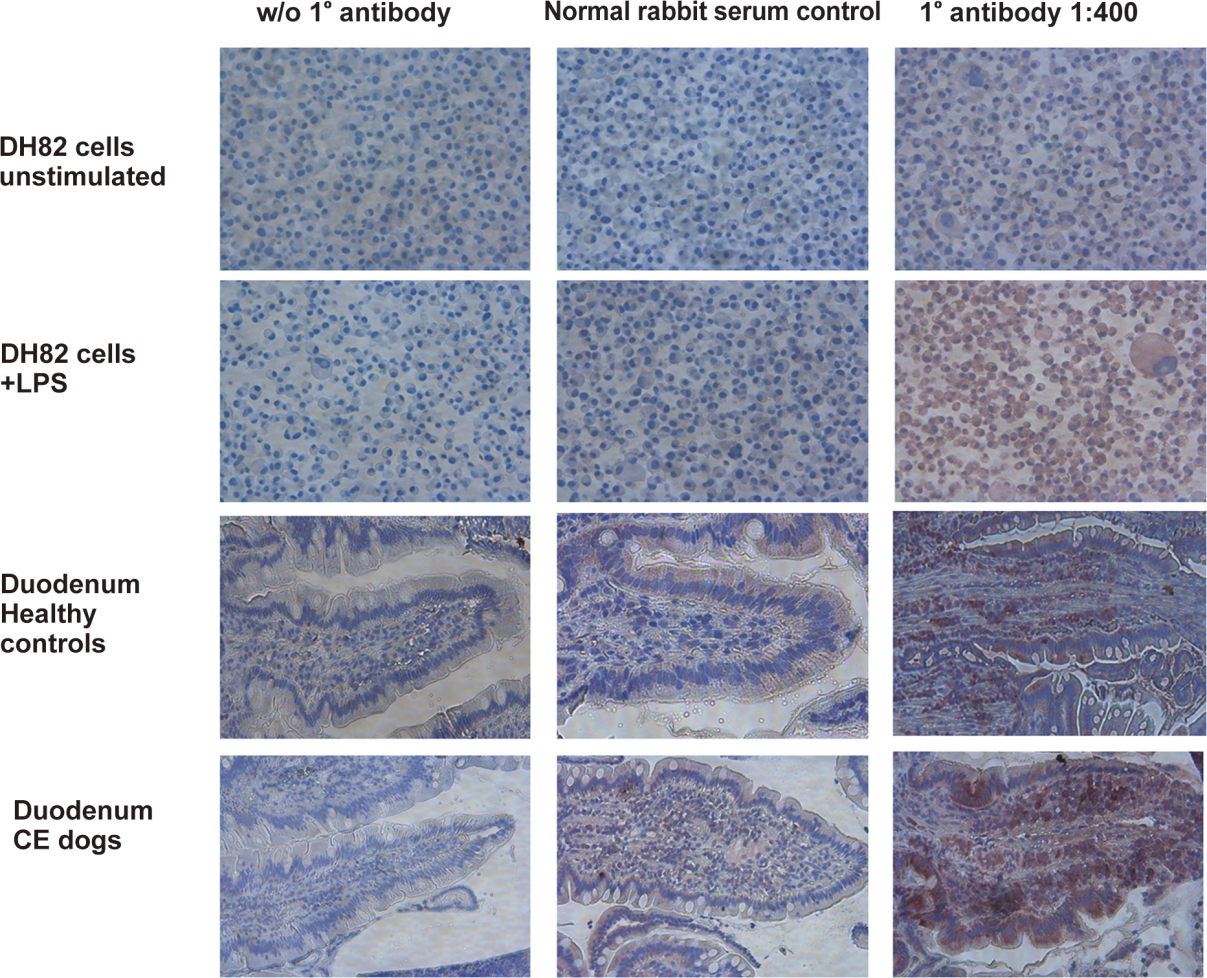
Immunohistochemistry for canine Interleukin-1β in the canine duodenum. Purple staining denotes positive cells (VIP-staining). Columns represent the tissue incubated with either the no-antibody control (diluents only; control serum or antibody at 1:400). Rows represent different types of tissues. DH82 cells serve as a negative (unstimulated) and positive (stimulated with lipopolysaccharides = LPS) control tissue. Duodenum control are samples from healthy dogs; CE = tissue from dogs with food-responsive chronic enteropathy.

**Fig 6:**
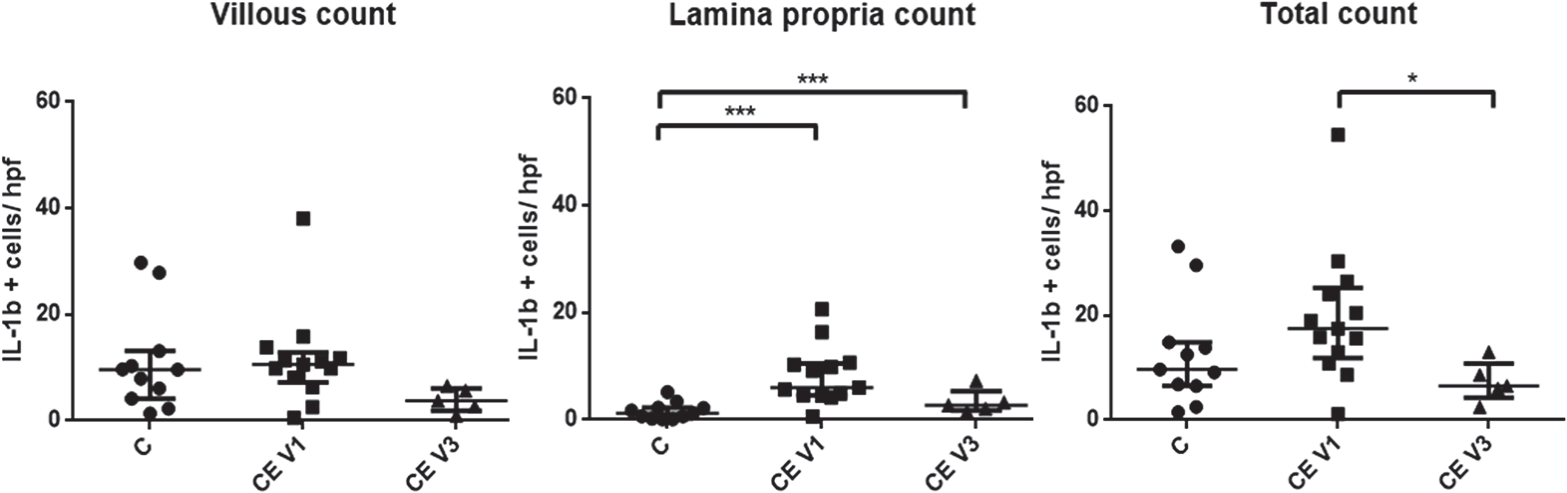
Averaged counts of cells staining positive for IL-1β in canine duodenal sections with immunohistochemistry. (A) Total number of cells positive for IL-1β as assessed by immunohistochemistry in healthy dogs, dogs with chronic enteropathy before treatment (V1 = visit one before dietary intervention) and after 6 weeks of treatment (V2 = visit two, 6 weeks into dietary intervention). (B) Number of IL-1β positive cells in the villous region. (C) Number of IL-1β positive cells in the lamina propria. * p < 0.05, *** p < 0.001.

We next assessed the presence and localisation of IL-1β protein (as one of the main “outputs” of inflammasome activation) in canine duodenal samples by immunohistochemistry (IHC). In detail, there were samples from healthy dogs (n = 12), archived samples from CE dogs (n = 11; these dogs histologically had lymphoplasmacytic or mixed enteritis and were diagnosed with idiopathic IBD) and samples from the prospective clinical trial mentioned below (these dogs were all diagnosed with food-responsive chronic enteropathy; 9 samples from visit 1/ before dietary intervention; 8 samples from visit 2/ after 6 weeks of hydrolyzed protein diet). Quantitative assessment of positively staining cells (Fig. 5) revealed that the total count of IL-1β producing cells was subjectively increased in CE dogs compared to controls (although not significant), and that the number of positive cells decreased significantly after treatment in the CE dogs (Fig. 6). Interestingly, this was not reflected by an increase of IL-1β positive cells in the villi, but mostly carried by an increase in positive cells in the lamina propria, which did not entirely return to baseline values after treatment (Fig. 6).

### Expression of inflammasome-related genes in the duodenum and colon is not altered by treatment for canine chronic enteropathy

To assess the effect of EF treatment *in vivo* on the inflammasome pathway in the intestine, as well as to compare to our *in-vitro* and *ex-vivo* data, we next examined the mRNA expression of inflammasome-related genes in duodenal and colonic samples from 12 dogs diagnosed with CE before (visit 1) and 6 weeks into treatment (visit 2) with a hydrolyzed antigen diet (the signalment of these dogs can be found in Table 1). Clinically, these dogs showed mild to moderate disease with a CCECAI median of 4.3 (sd 1.1) before treatment. Most dogs presented with mixed small and large intestinal signs (n = 6), 3 dogs had small intestinal diarrhea only, 2 dogs had chronic vomiting as the main presenting complaint and 1 dog had large intestinal diarrhea only. All of these dogs received gastroduodenoscopy and colonoscopy with multiple mucosal pinch biopsies. Their histopathological diagnosis for the duodenum was mild to moderate lymphoplasmacytic infiltration (n = 9), eosinopihilic inflammation (n = 2), and mixed inflammation (n = 1). For the colon, histological diagnoses included mild lymphoplasmacytic colitis (n = 5), normal tissue (n = 5) and eosinophilic colitis (n = 1). Histological scoring was performed using the WSAVA guidelines. Here the dogs had a median total score of 3 (range 1–5) in the duodenum and 2 (range 1–5) in the colon at the first visit. In addition to the diet, 7 of these dogs were randomly assigned to receive Synbiotic D-C (EF and prebiotics), and 5 to receive placebo. CCECAI decreased to a mean of 1.9 (sd 1.2) for all dogs combined with no significant difference between probiotic or placebo treatment. This decrease of clinical activity between the two visits was highly significant (p < 0.001), confirming these dogs to be food-responsive. Median total WSAVA histology scores was 1 (range 0–8) for the duodenum and 2 (0–4) for the colon after treatment. Linear mixed modelling of WSAVA scores with “visit” as the main effect revealed no significant difference between samples from before and after treatment. The only significant change was a decrease in inflammatory score from before to after treatment in the colon (p = 0.034), which translated into a significantly lower total colonic score (p = 0.012).

**Fig 7:**
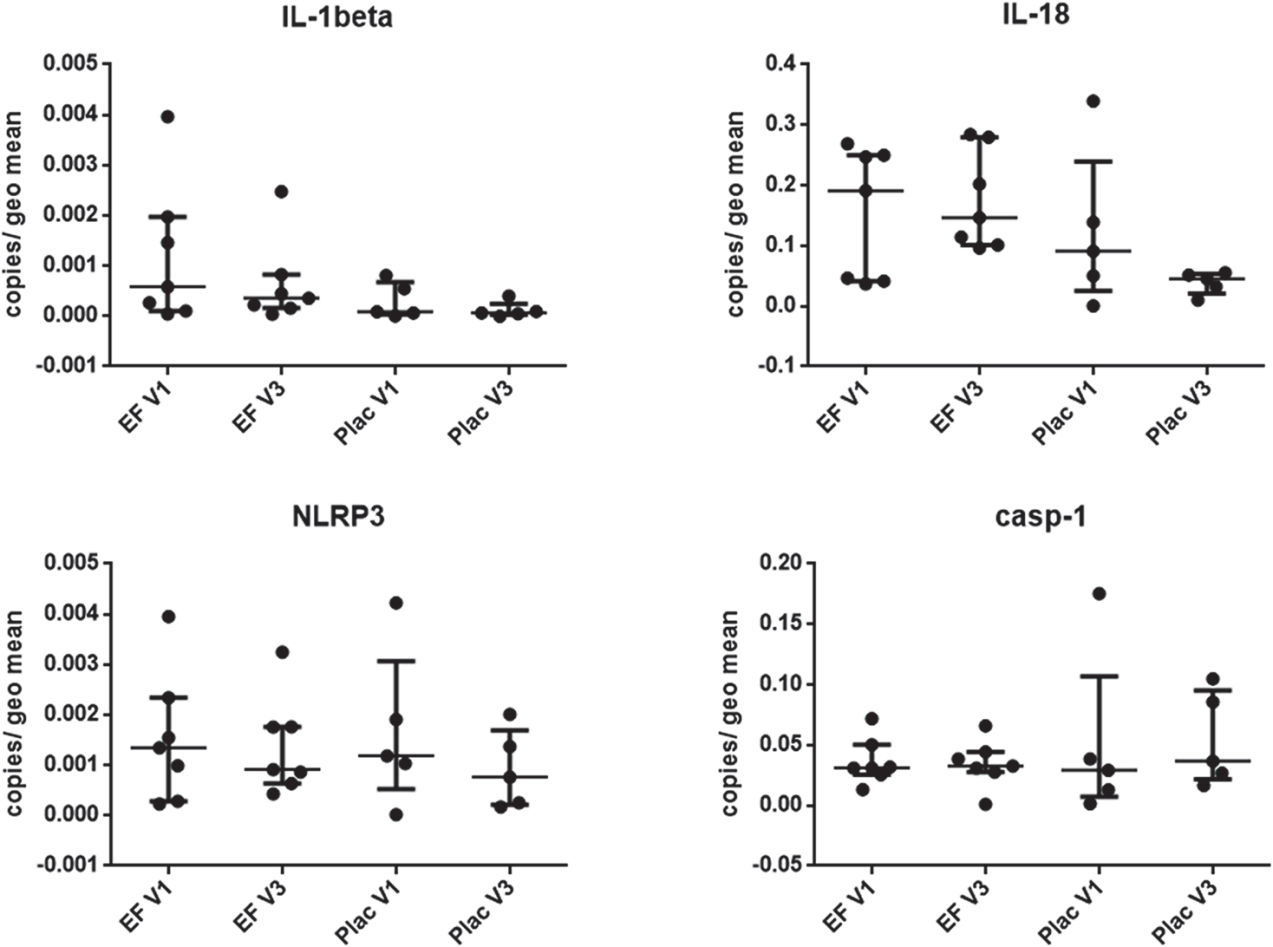
Relative expression of inflammasome-related genes in the duodenum of 12 dogs undergoing treatment for food-responsive chronic enteropathy. All dogs received a hydrolysed protein diet plus either Synbiotic D-C^©^ (*Enterococcus faecium [EF]* NCIMB 10415 & prebiotics) or placebo (Plac). Gene expression was determined from endoscopic pinch biopsies before (V1 = visit one) and after 6 weeks of treatment (V2 = visit two). Data are presented as median and interquartile range. IL = interleukin, casp-1 = caspase 1, NLRP3 = NOD-like receptor with pyrin domain 3.

**Fig 8:**
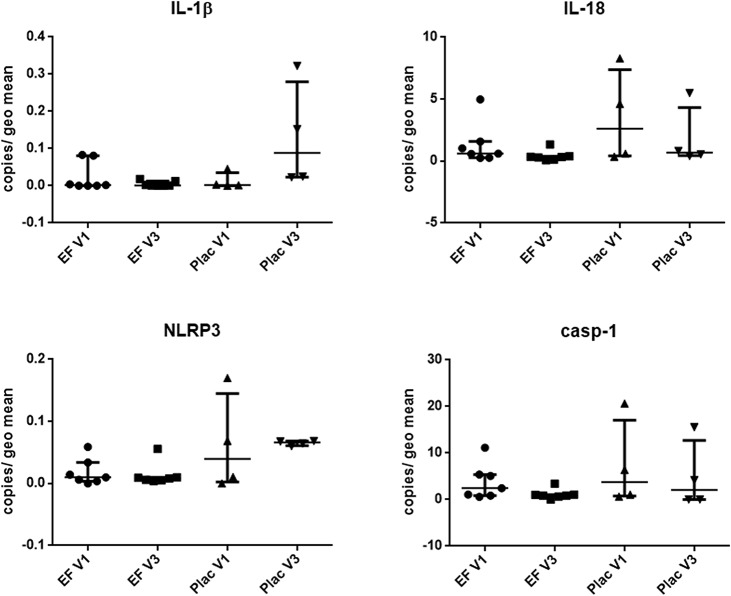
Relative expression of inflammasome-related genes in the colon of 12 dogs undergoing treatment for food-responsive chronic enteropathy. All dogs received a hydrolysed protein diet plus either Synbiotic D-C^©^ (*Enterococcus faecium [EF]* NCIMB 10415 & prebiotics) or placebo (Plac). Gene expression was determined from endoscopic pinch biopsies before (V1 = visit one) and after 6 weeks of treatment (V2 = visit two). Data are presented as median and interquartile range. IL = interleukin, casp-1 = caspase 1, NLRP3 = NOD-like receptor with pyrin domain 3.

In the duodenum, gene expression of IL-1β, IL-18, casp-1 and NLRP3 was not significantly different between dogs with probiotic or placebo treatment. Both IL-1β and NLRP3 were expressed at much lower levels than IL-18 and casp-1. Even though gene expression of IL-18 seems to subjectively reduce less in Synbiotic D-C treated dogs at visit 2 (Fig. 7) compared to the placebo group, this was not statistically significant. In general, it was observed that inflammasome-related genes were expressed at a much higher level in the colon than in the duodenum (both in healthy and CE dogs; Fig. 7 & 8), which was especially the case for casp-1. There was also no significant difference in IL-1β, IL-18 or NLRP3 gene expression before and at the end of treatment period in the colon (Fig. 8). Using linear mixed modelling, an interaction between treatment and visit was detected for casp-1 expression in the colon (p = 0.044), but there was no independent effect of disease status or treatment for this gene.

## Discussion

In the present study, we assessed how the application of EF to *ex-vivo* biopsies, or given as oral treatment, affects expression of inflammasome components in healthy dogs and dogs with CE. Due to the absence of more suitable antibodies, this was mostly done by assessing transcription levels using quantitative reverse-transcription PCR (qPCR), but also by IHC to assess protein production for selected components.

Only one other study has investigated so far the inflammasome output in dogs with CE so far. However, this was based on the assessment of the inflammatory cytokine IL-1β and the IL-1β receptor antagonist. No study has so far assessed different NLRs in healthy or diseased dogs. Here, no NLRP1 mRNA expression was detected in duodenal samples from healthy and CE dogs, which might imply that this gene does not play a major role in the innate immune mechanisms in the canine gut. On the contrary the absence of NLRC4 mRNA expression was not unexpected, as one other study has already described this gene as non-functional in the canine population [27]. Based on these results, we focused on the analysis of NLRP3, as at the time of the start of the experiments, no sequences for other NLRs such as NLRP6, 8, 9 or 14 were available. NLRP3 expression was significantly lower in samples from dogs with CE compared to healthy controls. This is in contrast to findings in experimental colitis in rodents, where NLRP3 is up-regulated [20]. It is thought that inflammasome activation occurs by increased permeability of the intestinal mucosal barrier, which leads to a higher bacterial load. However, inappropriately low NLRP3 expression could also be one of the causes of intestinal barrier instability and on-going inflammation, as NLRP3 has been shown to be important for the maintenance of intestinal mucosal integrity [22]. Thus, whether the low NLRP3 transcript levels seen here are a cause or result of intestinal inflammation, or simply play no role in canine CE pathogenesis remains unclear.

Caspase-1 is the major enzyme activated by NLR stimulation. Its purpose is to cleave the pro-forms of IL-1β and IL-18 into their active forms. Caspase-1 is hence assumed to be up-regulated on the mRNA and protein level in inflammatory conditions such as CE. The down-regulation of casp-1 mRNA in this study therefore contrasts findings in human IBD [16]. However, transcription levels do not provide information about enzyme activity levels. A casp-1 activity assay or the assessment of transcription of casp-1 subunits [28] would have been more informative, but was unfortunately not performed.

Having investigated the NLR component expression under ‘steady-state’ conditions in healthy dogs and dogs with CE, we next investigated the effect of an EF-stimulation of duodenal biopsies *ex-vivo*. Incubation with EF increased casp-1 transcript levels compared to stimulation with pure TLR ligands, but did not alter NLRP3 gene expression independent of disease status. Stimulation with other TLR ligands showed negligible effects on mRNA levels of the investigated inflammasome components apart from IL-18. This might not be entirely unexpected, as these are not direct stimulators of the inflammasome. NLRP1 and NLRC4 have a more select range of direct stimulators as far as known from experimental animal and human studies, whereas NLRP3 seems to have a wider array of possible stimulatory molecules. Flagellin signals through TLR5 and NLRC4 in humans and mice, but TLR5 gene expression was not changed in the *ex-vivo* stimulated samples and NLRC4 was not investigated for reasons mentioned above [27]. Nonetheless, increased TLR5 downstream signalling (without mRNA up-regulation) could result in a transcription bias towards NFκB-regulated cytokines, thereby shunting the inflammasome pathway (especially with no functional NLRC4 present), resulting in a lower casp-1 expression and/or activity.

Transcript levels of IL-1β and IL-18 were not significantly different between healthy and diseased dogs. This is again different to human IBD, where both IL-1β and IL-18 mRNA levels are highly up-regulated in intestinal biopsies [13–16, 29]. However, IL-1β protein production as assessed by IHC was increased in the lamina propria of CE dogs examined here compared to healthy control dogs, which could be an indication for increased casp-1 activity under certain inflammatory circumstances. This finding is similar to findings in human pediatric IBD [30]. Maeda et al. found similar results with regards to transcription of levels of IL-1β in dogs with CE [26]. However, these authors did not detect an increase of this cytokine’s protein levels, which could be due to the use of different techniques used in both studies (ELISA of homogenised tissue lysates *versus* IHC). Further disparity between mRNA and protein levels of IL-1β could stem from the fact that we did not assess IL-1β protein expression in IECs, even though some samples showed positive staining for IL-1β in IECs. It is known that human IECs in the steady state do not express IL-1β mRNA, but they can produce it upon stimulation (infection, inflammation) [31, 32]. In addition, the IL-1β IHC assay in the work presented here was performed with a polyclonal antibody against the human IL-1β protein; and even though the manufacturer of the antibody claimed cross-reactivity with the canine protein based on protein sequence homology, there is some question about the antibody’s specificity in canine tissues. Further testing of this antibody or the use of a monoclonal antibody would be needed to make a final conclusion about IL-1β protein production in canine CE tissues.

Similarly, the fact that IL-18 mRNA expression was not different between CE and healthy dogs has to be interpreted with caution. As mentioned in the introduction, both cytokines depend on activation by cleavage through caspase-1, thus transcript levels do not have to correlate with protein levels in the same tissue.

Unfortunately, measurement of inflammasome-related proteins in the biopsy culture supernatants was not successful. When assessing expression of inflammasome-related genes in samples from the clinical trial, it was interesting to note that inflammasome components are expressed generally to a higher level in the colon compared to the duodenum. Considering the fact that the bacterial load in the intestinal lumen is much higher in the large intestine compared to the small intestine, this was not unexpected. Overall, IL-18 was found to be expressed at much higher levels as any other cytokine investigated. This is concordant with another study [33]. There were no differences detected in any of the investigated inflammasome components at different time-points (before and after treatment) or with different treatments (Synbiotic D-C vs. placebo), even though especially IL-18 gene expression seems to undergo some subjective changes between visits, especially when stimulated *ex-vivo* with flagellin. Several samples from the clinical trial were also available for IHC assessment of IL-1β protein production in duodenal tissues. IL-1β-positive cells were found to decrease after treatment with an elimination diet, regardless of additional treatment with probiotic or placebo. This could be due to the fact that transcription levels of cytokines undergoing activation and possible post-translational modifications do not have to correlate with their protein production levels.

At this stage, an important role of the NLRs and their down-stream molecules in the development or maintenance of inflammation in canine CE cannot be excluded. Especially the reduction in IL-1β protein levels with dietary interventions in canine food-responsive CE seems an interesting target for future investigations. There also is the possibility to reduce IL-18 mRNA levels with treatment in CE. Additionally, determination of casp-1 enzymatic activity warrants further investigations.

Similarly, there is no firm evidence that probiotic treatment (*ex-vivo* or *in-vivo*) has any impact on the expression of the investigated inflammasome components in intestinal tissues from CE dogs. For *ex-vivo* studies, stimulation of intestinal tissues or selected intestinal cells with compounds more tailored towards inflammasome activation or blockage might be more appropriate. However, this might be improved by the selection of inflammasome-specific stimulants. For the clinical trial, there was no effect of EF on inflammasome gene expression. This might not exclude any beneficial effect of probiotics in this setting, but different bacterial strains, dosages or a different panel of genes to be investigated have to be considered.

## Methods

### Ethics statement for prospectively collected samples

Dogs with CE were recruited as part of a prospective clinical trial conducted at the Queen Mother Hospital for Animals (Royal Veterinary College, London, UK) investigating properties of the probiotic *Enterococcus faecium* NCIMB 10415 (EF). The study was performed according to the Animal Scientific Procedures Act (ASPA approval number 70/7393) and had received ethical approval by the Royal Veterinary College’s (RVC) Ethics and Welfare committee prior to the start of recruitment. Samples acquired from those dogs were processed in the molecular laboratories of the RVC’s Clinical Investigation Centre.

Control dogs consisted of Beagles from an experimental colony kept at the Justus-Liebig University (JLU; Giessen, Germany). Regional council approval had been received for obtaining fresh endoscopic intestinal biopsies from these dogs (no. 36/2011) according to the German Protection of Animals Act.

### Sourcing of samples from healthy and diseased animals used in the study

In the first part of this study, archived intestinal endoscopic biopsy samples from client-owned dogs with spontaneous CE (more specifically idiopathic IBD) and healthy dogs (collected as part of an unrelated study and kept at −80°C in RNA later) were used to compare baseline inflammasome gene expression. Clinical data or data of histological severity were unfortunately not available for these samples at the time this study was conducted. The signalment of these dogs can be found in Table 1. For the second part of this study (*ex-vivo* biopsy stimulation, see below), fresh endoscopic intestinal biopsies were collected prospectively from dogs participating in the clinical trial mentioned above (see Table 1). Samples from these dogs were also used to compare gene expression before and after treatment for food-responsive CE, as the trial involved a second endoscopy after 6 weeks of treatment. Therapy consisted of a standardised hydrolyzed protein diet for all dogs (Purina Veterinary Diets HA Hypoallergenic) and either Synbiotic D-C (EF 1 x 10^9^ CFUs, fructo-oligosaccharides, gum Arabic; Protexin Ltd., Somerset, UK) or placebo (maltodextran) in a double-blinded fashion. For all cases, the diagnosis of CE was based on the presence of appropriate clinical signs (vomiting and/or diarrhoea ± weight loss) of at least 3 weeks’ duration, exclusion of other causes of chronic gastrointestinal signs and the presence of lymphoplasmacytic and/or eosinophilic inflammation on histopathological examination of intestinal biopsies. Food-responsive CE was defined as improvement of clinical signs as assessed by the canine chronic enteropathy clinical activity index (CCECAI) while fed the standardised hydrolysed protein diet for 6 weeks.

Control dogs were deemed healthy based on the absence of clinical signs, normal physical examination and no abnormalities on routine haematology, serum biochemistry and intestinal histopathology.

### Isolation of total RNA and Reverse transcription

Endoscopic intestinal biopsies were homogenised in 350 μl RLT lysis buffer each (Qiagen, Manchester, UK), using 5 mm stainless steel beads (Qiagen) and the Mixer Mill MM300 tissue grinder (Retch, Leeds, UK). Total RNA was extracted using the RNeasy micro kit (Qiagen) as per manufacturer’s instructions, including an on-column DNAse treatment. Samples were eluted in 30 μl distilled water. RNA quantity and quality was assessed using the Eukaryote Total RNA Nano chip with the Agilent BioAnalyzer (Agilent Technologies, Wokingham, UK), and only RNA with a RIN number of > 6 was used. Using this assay, no DNA contamination could be detected in any sample.

Reverse transcription was performed using the iScript cDNA synthesis kit (Bio-Rad, Hemel Hampstead, UK), which uses a mixture of oligo-dT and random nonamer primers.

### Standard polymerase chain reaction to detect presence of NLR mRNA

Polymerase chain reaction (PCR) was performed using the Immolase taq polymerase (Bioline, London, UK) as outlined by the manufacturers. Reactions were performed in a final volume of 25 μl with 1 μl of template, 800 pmol of primers and 2.5 mM MgCl_2_. Cycling was performed at the following conditions: 95°C for 7 min for enzyme activation, then 35 cycles of 95°C for 30 sec (denaturation), annealing for 30 seconds at 55°C, 72°C for 1 minute (extension) and a final extension step at 72°C for 7 minutes. Primers were based on published gene sequences sourced from Genbank (http://www.ncbi.nlm.nih.gov/genbank/) and/or ENSEMBL (www.ensembl.org) servers (versions 68–70) (see Table 2). They were designed using the Primer3 online tool (frodo.wi.mit.edu/) with the default specifications and manufactured by Eurofins MWG Operon (Ebersberg, Germany). Primer sequences were checked for target specificity by using Basic Logic Alignment Search Tool (BLAST; http://blast.ncbi.nlm.nih.gov/Blast.cgi *)*. *PCR products were visualised on a 1*.*5% agarose gel*.

**Table 2:** Internal primers used to amplify parts of genes connected to the inflammasome.

**Gene**	**product size**	**Forward**	**Reverse**	**Accession number**
Caspase-1	253	CGACAGACAG CTGGACACAT	ATCTGGGCTTT CACATCTGG	NM_001003125.1
IL-1 beta	440	GCAGTACCCG AACTCACCAG	ACATTTTCCCC ATTGAGGTG	NM_001037971.1
IL-18	240	GAGGATATGC CCGATTCTGA	ATCATGGCCTG GAACACTTC	XM_005619483.1
NLRC4	365	TGAGCAGCAG TGTTTTCACC	TGGCTTCCATA TCCTCCCTA	NC_006599.2
NLRP1	363	CCTCTTTGGC CTTCTGAGTG	CTGAACAGAGC CACACTGGA	XM_00546567.4
NLRP3	207	GCAATGCTCT TGGAGACACA	AGAGCAGCATG ACCCCTAGA	XM_00843284.2

### Reverse-transcriptase quantitative PCR for the detection of inflammasome-related gene expression

To obtain positive controls suitable for subsequent quantitative PCR (qPCR) analysis, parts of the canine sequences for the genes of interest and 3 reference genes were cloned as described previously [34]. These plasmids were used in a 10-fold dilution (10^7^ molecules μl^−1^ to 10^1^ molecules μl^−1^) to create a qPCR standard curve for each gene and run and to assess assay efficiency. Each qPCR reaction was performed in 20 μl, contained 200 nM of each primer and 1 μl of cDNA in addition to SsoFast Evagreen Supermix (Bio-Rad). Characteristics of the primers used can be found in Table 2. Cycling conditions consisted of an enzyme activation step at 95°C for 30 seconds, followed by 40 cycles of 95°C for 10 seconds, annealing at 55°C for 10 seconds and elongation at 65°C for 10 seconds. An additional 5 seconds melting step was included before each plate read depending on the melting curve analysis of the respective PCR products (to melt primer dimers). Each reaction was carried out in triplicate. Melting curves were generated for each run to ensure a single amplicon had been produced.

Gene expression was quantified by averaging the triplicate absolute gene copy number for each biological sample for all genes, following normalisation of the expression of each target gene to the geometric mean of the three reference genes (glyceraldehyde 3-phosphate dehydrogenase [GAPDH], TATA-box binding protein [TBP], succinate dehydrogenase complex subunit A [SDHA]), as described recently [34, 35]. These genes have been reported to be most stable in canine duodenal tissue [36], and a minimum of 3 reference genes was selected to adhere to the MIQE guidelines [37].

### *Ex-vivo* stimulation of duodenal biopsies with TLR ligands and Enterococcus faecium

*Ex-vivo* culture of duodenal biopsies was performed using a previously described protocol [38] with minor modifications: Eight duodenal pinch biopsies obtained during duodenoscopy were immediately transferred into ice cold culture medium (RPMI 1640 + glutamine, Gibco, Paisley, UK) with 100 U ml^−1^ penicillin and 100 μg ml^−1^ streptomycin (PAA, Somerset, UK) and carefully rinsed by decanting 3 times. Penicillin/ streptomycin were added in order to control the microbiota attached to the samples, but as soon as probiotic bacteria were added, RPMI medium without antibiotic additives was used. Biopsies were carefully transferred in pairs to a well of a 24-well flat-bottomed plate (Nunc) holding 900 μl of medium. One-hundred μl of one of the different bacterial stimulants (see below) were added. This included Toll-like receptor (TLR) ligands (Invivogen, San Diego, USA) to simulate stimulation with well-defined single bacterial ligands, as well as live cultures of EF. TLR ligands were added to reach the following final concentrations (as determined by a pilot study; data not shown): Lipopolysaccharide (LPS; *Escherichia coli 0111*:*B4;* 1 ng ml^−1^, TLR4 ligand), Pam_3_CSK_4_ (100 ng ml^−1^; TLR1/2 ligand) and recombinant flagellin from *Salmonella typhimurium* (1 μg ml^−1^; TLR5 ligand). EF was used at a concentration of 1 x 10^7^ cfu ml^−1^. PBS was used as a negative control. Plates were incubated for 5 hours at 37°C and 5% CO_2_. Biopsies were then transferred to 1.8 ml cryotubes holding 1 ml of RNA later (Ambion, Huntingdon, UK), kept at 4°C for 24 hours and transferred to −80°C for storage until further use. Culture supernatants were harvested, aliquoted and stored at −20°C until further use.

### Immunohistochemistry for Interleukin-1β

Slides were prepared from duodenal samples from healthy and CE dogs from formalin-fixed, paraffin-embedded tissue blocks originally created as part of the routine histopathological assessment. Slides were deparaffinised using histology grade xylene (Sigma-Aldrich, Dorset, UK) for 3 x 5 min and rehydrated using descending concentrations (100%, 70%, 50%, 30%) of molecular grade ethanol (Sigma-Aldrich) for 3 min each. Slides were then rinsed for 5 min in tap water. Heat-induced antigen retrieval was performed in 0.01 M sodium citrate, pH6 (“antigen unmasking solution low pH”, Vectorlabs, Peterborough, UK) using a standard 800 W microwave on full power for a total of 13 minutes. From here on, a washing step was performed in-between each incubation step by rocking the slides gently in a tray holding 250 ml of wash buffer (1 X TBS [Fisher Scientific, Loughborough, UK] with 0.1% Tween 20 [Sigma-Aldrich]). Blocking endogenous peroxidase activity was performed using 0.3% hydrogen peroxide (Sigma-Aldrich) in methanol (Sigma-Aldrich) for 30 min at room temperature. Protein blocking was performed using 2.5% horse serum (Vectorlabs) with 1% bovine serum albumin (Sigma-Aldrich) for 1 h at room temperature. After incubation with the protein block, slides were not washed, but the liquid only tipped off. The primary antibody, a rabbit-raised polyclonal IgG antibody against a 17 kDa recombinant peptide from the human IL-1β protein (the cleavage site generated by caspase-1; cat# ab34837, Abcam, Cambridge, UK), was applied to the slides immediately at a concentration of 1:400 (diluted in 1X TBS + 1% horse serum + 0.1% Tween 20 + 0.1% Triton X-100 [Vectorlabs]). Incubation with the primary antibody was performed for 6 hours at 4°C. The ImmPRESS polymer-based horse-radish peroxidase kit (Vectorlabs) was used for detection of the target as by the manufacturer’s instructions: Sections were incubated with the ImmPRESS anti-rabbit reagent for 30 minutes at room temperature. The substrate used was Very Intense Purple (VIP; Vectorlabs) as instructed by the manufacturer: slides were incubated with the freshly made substrate solution for 2 minutes, and then rinsed in tap water for 5 minutes. Counterstaining was performed with haematoxylin for 5–7 seconds, followed by rinsing in tap water and dehydration using ascending concentrations of ethanol (reverse order as for the rehydration above). Slides were mounted with Immumount (Vectorlabs) and covered with a cover slip. Slides sine 1° antibody and slides where the 1° antibody was substituted with normal rabbit serum (1:1000; Vectorlabs) were included simultaneously as controls. Positive controls included slides from pelleted DH82 cells (a canine macrophage/ monocyte cell line) which had previously been stimulated with 1 μg/ml LPS for 3 hours to provoke IL-1β production; as well as unstimulated pelleted DH82 cells as negative tissue controls (see Fig. 5).

### Statistical analyses

Statistical analysis was done using IBM SPSS statistics version 19.0. To compare gene expression data between healthy and diseased duodenal tissue at baseline, Mann-Whitney U test was performed. To assess the effect of different stimulations in the *ex-vivo* assay between healthy and diseased samples, linear mixed modelling with “treatment” and “disease status” as main effects was performed (hence all *ex-vivo* treatments could be compared to each other as well as between samples from dogs with or without disease). A similar approach was taken for the evaluation of differences in gene expression before and during treatment for CE, with probiotic or placebo “treatment” as well as time of visit (visit 1 = before dietary treatment; visit 2 = after 6 weeks of dietary treatment) set as main effects. ELISA data from *ex-vivo* culture supernatants were analysed using one-way ANOVA. Finally, cell counts from immunohistochemistry (healthy dogs vs. CE dogs before treatment vs. CE dogs after treatment) were compared using Kruskall-Wallis test.
